# Supplementary material for: Are carbon emissions trading and green financial instruments synergistic? -Comprehensive quantitative research based on content analysis
Source: PLoS One. 2024 Mar 7;19(3):e0298601. doi: 10.1371/journal.pone.0298601 (PMC10919601; doi:10.1371/journal.pone.0298601)
Supplement: S1 Data — (ZIP) [file pone.0298601.s001.zip › Data/地级市Z_F工作报告.zip!/║ú─╧/╚2╤╟╩╨/└┤╫╘╣1⁄2╓┌║┼í╛┬φ┐╦╩2╛▌═°í┐_2010.docx]

各位代表：

现在，我代表市人民政府向大会作工作报告，请予审议，并请市政协各位委员和其他列席人员提出意见。

2009年主要工作与发展回顾

2009年以来，国际金融危机的影响进一步加深，国内外发展的不确定因素较多。面对严峻的考验，市政府坚持以科学发展观为指导，在市委的正确领导下，在市人大、市政协的监督支持下，认真贯彻落实“保增长，扩内需，调结构”的一系列调控措施，积极应对挑战，努力化危为机，扎实推进工作，经济总体上保持了平稳较快发展的态势，社会事业全面进步，人民生活不断改善，顺利完成了市五届人大四次会议确定的年度任务，取得了较好的发展成绩。

――经济平稳较快发展。初步核算，2009年全市生产总值174.85亿元（不含农垦，下同），同比增长17.2%（按可比价格计算，下同）；按户籍人口计算，全市人均生产总值36065元，增长14.8%。全社会固定资产投资总额211.4亿元，同比增长39.5%。社会消费品零售总额50.9亿元，同比增长25.3%。

――发展质量有所提升。全市地方一般预算收入26.7亿元，同比增长26.7%；其中，税收收入23.55亿元，增长29%。税收收入占GDP的比重由2008年的12.1%提升到13.5%。截至去年12月底，全市金融机构本外币各项存款余额、贷款余额分别比去年年初增长70.1%、53.1%，金融机构整体盈利同比增长16.2%。产业结构进一步优化，第一、二、三产业分别实现增加值27.34亿元、40.36亿元、107.15亿元，同比分别增长7.9%、19.7%、18.9%，对经济增长的贡献率分别为8%、25.9%、66.1%。第三产业税收收入19.12亿元，占地方税收收入的81.2%。

――民生事业取得新进步。加快实施《三亚市2008―2013年重点民生项目发展规划》，城乡居民增收、就业、社保、住房保障、教育、医疗卫生、文化体育、公共安全、生态环保、新农村建设等方面的财政投入力度进一步加大，全年拨付重点民生项目资金25亿元，同比增长42.2%。城乡居民收入进一步提高，生活进一步改善，全市城镇居民人均可支配收入15233元，同比增长13.1%；农民人均纯收入5620元，增长8.3%。

――生态环境质量保持优良。节能减排工作进一步加强，完成了省里下达的单位GDP能耗指标以及化学需氧量、二氧化硫排放量指标。积极开展生态环保和环境治理工作，全市环境空气质量、水环境质量总体状况良好，2009年末全市森林覆盖率达到68%，建成区绿化覆盖率45.2%、绿地率41.5%、人均公共绿地面积18.97平方米。

一年来，我们切实加强了以下六个方面的工作：

（一）积极采取有效措施，应对危机保增长。及时出台了《关于促进经济平稳较快发展的决定》和配套实施细则，加大了财政金融对经济发展的支持力度。2009年共安排产业扶持及重点项目建设资金8.4亿元，对非政府投资重点项目给予财政补贴，对重点旅游项目建设贷款给予财政贴息，实施航空补贴、旅行社奖励等政策，保障了大型活动、旅游促销、房地产推介展销活动经费，加大了对中小企业的信贷投放力度。积极改善投资环境，全力抓好项目建设。新增中央投资方面，截至去年年底，我市新增中央投资项目共四批次12类24个项目，总投资6.96亿元，累计完成投资6.17亿元，12个项目已建成投入使用。重点项目方面，2009年共有84个重点项目实施建设，全年完成投资141.2亿元。其中，红塘湾国际高尔夫球场、槟榔田洋设施农业、09年新农村道路建设、市人民医院改扩建一期工程等项目已基本完工，凤凰国际水城、时代海岸、三亚绕城高速公路、琼州学院三亚校区和西部供水工程等项目加快推进。海棠湾开发建设态势强劲，2009年累计完成固定资产投资40.27亿元，道路、供水、污水处理等市政配套设施进一步完善，开维凯宾斯基、中港万丽等酒店项目顺利推进，国家海岸形象进一步提升。

（二）推进产业结构调整，着力转变发展方式。面对金融危机和甲型H1N1流感等不利因素的影响，加大了国内旅游宣传促销力度，保障了旅游产业总体平稳发展。2009年全市共接待旅游过夜人数669.1万人次，同比增长10.7%；旅游收入103.8亿元，增长14%。积极组织房地产促销活动，切实落实各项优惠政策，全年房地产投资97.9亿元，同比增长28.5%；商品房销售面积114.5万平方米，增长48.2%。大力发展设施农业，切实落实各项惠农政策，推动农业结构调整，全年农业完成总产值40.4亿元，同比增长8%。充分发挥投资需求的带动作用，建筑材料、供电量等主要工业产品产量大幅增长，全年工业完成总产值33.8亿元（不含中海油），同比增长24.2%。加快了商贸服务体系建设和商贸产业发展，认真实施汽车、家电、农机下乡和减征小排量车辆购置税等政策，全市消费品市场持续活跃。积极推动创意产业、文化产业等新兴产业发展，编制完成了《三亚市科技创意产业发展规划》，加快了创意产业园、市高新技术产业园基础设施建设，积极开展项目招商，创意产业园已有中兴通讯等10家企业入园；成功举办了世界精英模特大赛、世界沙滩排球巡回赛三亚公开赛等活动，加快推进三亚市体育中心等基础设施建设，继续深化文化体制改革，进一步改善了文化发展环境。在推进产业结构调整的同时，加快了节能减排工程建设，切实落实工作任务和责任，增强了可持续发展能力。污染源自动监控中心已建成，红沙污水处理厂升级改造工程已全面建成并投入运行，市生活垃圾卫生填埋场已投入试运行，城市污水管网改造工程、城市生活垃圾收集转运工程、亚龙湾区域集中供冷节能工程等项目进展顺利。积极开展海洋环境整治和海洋资源保护活动，大力推进山体植被恢复、海湾河道治理、重要水源保护等工程，生态环境质量继续保持全国一流。

（三）继续深化改革开放，努力增强发展活力。59家国有企业关闭退出工作取得阶段性成果，首批25家中已有20家完成了关闭退出的法定程序。扎实推进医药卫生体制改革，制定了《三亚市深化医药卫生体制改革近期重点工作实施方案（2009-2011年）》，率先在全省实施国家基本药物制度。集体林权制度改革已全面铺开并取得阶段性成果。农垦中小学校移交地方管理工作已基本完成，农场属地管理工作有序推进。充分利用“珠洽会”、“厦交会”等平台，积极组织招商活动，拓展合作空间。海南首家市内免税店已在我市试营业。大力开展对外交流，与佛得角共和国萨尔市结为友好城市，开通了三亚到哈萨克斯坦阿拉木图的直航包机，第四届中国邮轮产业发展大会暨首届国际邮轮博览会在三亚成功举办。

（四）加快城乡建设步伐，夯实三亚发展基础。积极推进新一轮城市总体规划以及城市综合交通规划、老城区综合交通整治规划等专项规划编制工作，进一步加强城市基础设施建设，吉阳大道、鹿回头海景大道等道路已建成通车，城市电网、给排水系统、天然气管网进一步完善。加快了旧城改造进度，城中村改造规划工作有序开展，月川江山片区改造已全面启动，榕根社区旧城改造前期安置用地工作已完成，下洋田旧城改造加快推进。不断强化城市管理，进一步完善“网格化”管理责任制，实现“定人、定岗、定责”。在严厉打击违法建设、非法营运等行为的同时，有针对性地推行疏堵结合的管理方式。大力开展环境综合整治活动，各项指标达到国家卫生城市标准要求，成为我省首个国家卫生城市。全面完成了新农村规划设计工作。大隆灌区西干渠、病险水库除险加固、妙林田洋农田整治等工程进展顺利，“十一五”农村公路规划建设任务已提前完成，新开通了多条农村公交线路。农技110服务站实现全市覆盖，农村沼气、民房改造、饮水安全、扶贫攻坚等工作取得新成效。

（五）大力加强社会建设，千方百计改善民生。就业方面，不断完善和切实落实就业优惠政策，积极开展各种就业专项活动，全市城镇新增就业岗位30220个，农村富余劳动力实现转移就业18413人，下岗失业人员实现再就业4102人，城镇登记失业率1.42%。社保方面，出台了《三亚市城镇老年居民基本养老保险实施办法》等文件，新型农村社会养老保险试点工作有序推进，在全省率先建立了统一的覆盖全体城乡居民的基本医疗保险制度。加大了城乡特困群众救助力度，全市低保覆盖面达到100%，城乡低保对象及困难群众的生活保障水平进一步提高。居住方面，积极建立健全住房保障制度，廉租住房、经济适用房等保障性住房建设顺利推进，自2008年以来已建成廉租住房2469套、经济适用房4354套，发放住房租赁补贴1013万元。教育方面，积极开展新一轮中小学布局调整和规划建设工作，大力改善学校办学条件，中小学校舍安全工程、教育移民工程进展顺利。三亚中等职业技术学校二期工程已基本完工。成立了学生资助管理中心，进一步扩大家庭经济困难学生资助覆盖面并提高了相关补助标准，义务教育“两免一补”、中职学校“三免一补”和高中学生免学费等政策得到较好落实。医疗方面，市中医院二期工程和解放军总医院海南分院等项目建设快速推进。完成了西岛社区卫生服务站建设，社区卫生服务网络覆盖面达到100%。进一步强化公共卫生服务，突出抓好甲型H1N1流感等疾病防治工作，无重大疫情发生。社会和谐方面，精神文明建设取得新进步，圆满完成了各项重大活动的安保工作，社会治安综合治理成效显著，荣获全国综治工作最高荣誉“长安杯”。食品药品安全保障有力，安全生产工作进一步加强，信访渠道进一步畅通，社会保持和谐稳定。

（六）推动体制机制创新，进一步提升行政效能。以转变政府职能、理顺职责关系、明确和强化责任为主要目标，调整和完善政府机构设置，明确市政府设置工作部门31个、部门管理机构6个、议事协调常设机构1个。目前各部门和机构的“三定”方案已基本完成。省里下放的行政管理事项平稳运行，政务服务中心建设进展顺利，行政审批制度改革进一步加快，我市政府各部门和事业单位保留的行政审批事项已压缩至450项。出台了《三亚市机关效能告诫暂行办法》、《三亚市行政过错责任追究暂行办法》，加强了工作督查、督办，结合推行首问责任制、岗位责任制、重大事项跟踪督办制，提升了政府行政效能。去年市政府常务会议议定事项共190项，其中183项已落实完毕。进一步规范重要岗位和重点领域权力运行制度，切实加强财政投资项目评审管理，加强审计监督和行政监察，积极开展反腐倡廉“制度执行推进年”活动，对房地产开发领域违规变更规划、调整容积率等问题进行专项整治。切实解决损害群众利益的突出问题，政府廉政建设取得实效。严格执行市人大及其常委会的决议、决定，定期向市政协常委会通报政务情况，自觉接受市人大和市政协的监督，认真办复市人大代表建议、批评和意见68件、市政协委员提案124件。积极加强同各民主党派、工商联、人民团体和无党派人士的联系，进一步规范政务公开工作，主动接受人民群众的监督。

一年来，我市新闻出版、民族宗教、统计、外事、侨务、人口计生、气象、防震减灾、档案、地方志等工作取得了新的成绩，工会等工作进一步加强，妇女儿童、老龄、红十字和残疾人等事业健康发展，国防动员、民兵预备役工作不断进步，拥军优属、拥政爱民活动深入开展，军政军民团结进一步巩固。

各位代表，过去的一年，在复杂的形势和严峻的考验面前，全市人民团结奋斗、顽强拼搏、共克时艰，我市经济社会发展取得了令人振奋的成绩。这得益于省委、省政府和市委的正确领导，得益于市人大、市政协、各民主党派、工商联、无党派人士以及各人民团体的监督支持，得益于中央和省驻三亚各单位、驻三亚部队、武警官兵、公安司法干警的参与配合，得益于海内外朋友的关心、支持和帮助。在此，我谨代表市人民政府，向各位人大代表、政协委员和各民主党派、工商联、人民团体，向奋斗在各条战线上的全市广大干部群众，向所有关心和支持三亚发展的同志们、朋友们表示衷心的感谢和崇高的敬意！

回顾过去的一年，在看到成绩的同时，我们也清醒地认识到，在发展的道路上仍然存在一些困难和问题：一是经济增长的基础还不稳固。尽管去年我市的整体投资规模保持了稳定增长，但主要是依靠政府投资支撑，民间投资增长缓慢，比政府投资增幅低17.3个百分点。海外游客接待量大幅下滑，全年接待海外游客31.8万人次，同比下降37.9%。外贸形势依然严峻，全年全市进出口总值4730万美元，同比下降38.1%；其中，出口4650万美元，下降30%。产业结构仍需进一步优化调整，创意、文体、海洋、商贸等产业发展仍需加快。二是劳动力供求结构性矛盾依然存在。一方面，技术工人和旅游服务业人员短缺，企业苦于“用工难”；另一方面，部分农村富余劳动力以及城镇失业人员观念陈旧、文化水平不高、就业技能单一，苦于“求职难”。三是民生事业发展仍需加快。各项社会事业欠账较多，征地拆迁工作有待完善，城乡发展差距仍然较大，一些涉及群众切身利益的问题急需解决。四是政府自身建设有待进一步加强。政府行政效能和服务水平与经济社会发展的需求和人民群众的要求还有较大差距，形式主义、官僚主义、行政不作为、乱作为等现象依然存在，效率低、“事难办”的问题尚未得到根本解决。五是城市功能和面貌与国际性热带滨海旅游城市的定位仍有较大差距。随着旅游产业和经济社会的不断发展，城市交通、环境卫生、社会治安、旅游市场秩序等方面的问题逐步暴露并日益凸显，城市基础设施建设和旧城改造亟待加快，城市功能和配套服务亟待完善，城市管理水平亟待提高。去年年底，国际旅游岛建设正式上升为国家发展战略，这给三亚带来了新的发展机遇，同时也使三亚再次成为社会各界关注的焦点。今年春节黄金周，三亚旅游市场人气旺盛，游客接待量大幅增长，但交通拥堵、出租车拒载、环境卫生脏乱差、海鲜排档欺客宰客等现象也引发了大量投诉和反映，引起了游客和媒体的强烈关注和批评。这些问题固然是发展中的问题，也并不能代表三亚的整体形象，但在服务设施建设滞后和管理缺失上，市政府应当承担不可推卸的主要责任。在这里，作为市长，我向各位代表和全市人民检讨！对于存在的问题和各方面的批评意见，市政府将敢于正视，并采取有效措施努力改进和解决。广大游客、人民群众和社会各界的关注和监督，正是对政府工作最有力的鞭策。

2010年发展目标和主要任务

2010年是实施“十一五”规划的最后一年，也是国际旅游岛建设的开局之年，做好今年各项工作，对保持经济社会良好发展态势、为“十二五”规划启动实施奠定坚实基础具有十分重要的意义。

今年我市所面临的发展形势总体上将好于去年。从全国的环境来看，去年扩大内需政策效果明显，我国经济运行已呈现出企稳回升的态势。今年中央将继续实施积极的财政政策和适度宽松的货币政策，推动经济发展方式转变和经济结构调整，扩大居民消费需求，这将为三亚发展提供有利的政策环境。从我省的形势来看，经过多年来的平稳较快发展，全省经济基础更加牢固，产业支撑力不断增强，环境、资源和区位优势日益显现。去年年底，国务院正式发布了《关于推进海南国际旅游岛建设发展的若干意见》，中央政策支持力度空前加大，给海南和三亚带来了重大的历史机遇。从我市的自身情况来看，自1998年以来三亚经济已连续12年实现两位数增长，经济发展的质量和效益不断提高，城乡基础设施更加完善，发展的基础进一步巩固。目前，三亚仍处于重要的发展战略机遇期，随着海棠湾等重点片区、重大项目开发建设和民生事业发展的不断加快，支撑我市发展的动力将进一步增强。当然，受国际金融危机的影响，世界经济仍处于艰难境地，国内外发展环境中仍然存在一些不可预见的风险，我市的国际旅游和外贸出口仍将受到较大的影响，我们也必须做好应对困难和挑战的准备。总之，2010年我市发展将面临一个机遇与挑战并存、总体上有利于发展的环境。我们一定要切实增强发展信心和风险意识，把握好战略机遇，积极应对各种挑战，努力保持和巩固来之不易的良好发展局面。

根据省委、省政府的总体要求和市委的部署，2010年市政府工作的总体思路是：全面贯彻党的十七大和十七届三中、四中全会以及省委五届六次、七次全会精神，以邓小平理论和“三个代表”重要思想为指导，深入贯彻落实科学发展观，以强市富民为目标，紧紧围绕国际旅游岛建设，继续推动经济平稳较快发展，加快转变经济发展方式，进一步深化改革开放，着力提升城市品质，全力改善人民生活，确保完成今年和“十一五”规划各项目标任务，努力打造中外游客的度假天堂和三亚人民的幸福家园。

根据上述思路，综合考虑各方面的因素，2010年我市经济社会发展的主要预期目标是：地区生产总值增长13%；固定资产投资增长25%；社会消费品零售总额增长18%；地方财政一般预算收入增长18%；全市接待过夜游客人数增长12%；万元GDP能耗下降1.4%；城镇居民人均可支配收入增长9%，农民人均纯收入增长8%；当年新增就业岗位2万个以上，城镇登记失业率控制在3%以内；人口自然增长率控制在10.7‰以下。为实现上述目标，今年要重点抓好以下八个方面的工作：

一、努力打造旅游度假天堂，争当国际旅游岛建设的排头兵

国际旅游岛建设已上升为国家发展战略，我们要积极主动做好政策对接，加快体制机制创新，推进旅游要素转型升级，全面提升旅游管理和服务水平，努力打造中外游客的度假天堂，在国际旅游岛建设中发挥重要作用。

（一）科学编制和完善相关规划。省里将在今年上半年完成国际旅游岛建设发展规划纲要并对现有规划进行修编完善，我们要在这一框架体系内，尽快出台国际旅游岛建设三亚行动方案，积极做好“十二五”规划编制，加快完成新一轮城市总体规划，并结合国际旅游岛建设进一步完善三亚战略规划体系，加快修编天涯海角、南山、大小洞天、鹿回头等重点景区规划，加强各项规划的有机衔接，控制好开发、建设、发展的强度、节奏与时序，为三亚更好地融入国际旅游岛建设提供科学的依据。

（二）积极主动做好政策对接工作。充分发挥区域旅游中心城市的优势，围绕国际旅游岛的六大战略定位，加快体制机制创新，加强与国家和省有关部门的沟通衔接，争取在我省各市县中较快出台具体的实施办法、操作方案，创造条件使中央给予我省的投融资政策、财税政策、土地政策、开放政策等各项政策尽早在三亚落实和用好。

（三）突出抓好海棠湾等片区建设。在海棠湾，要进一步加快市政配套设施和开维凯宾斯基、中港万丽等酒店建设进度，开工建设奥林匹克湾、沃顿财富学院和炎黄艺术中心等项目；在亚龙湾，要重点推进二期开发，加快三亚太阳湾度假区、瑞吉度假酒店等项目建设，推动洲际滨海大酒店、美高梅度假酒店尽早动工；在红塘湾，要加快启动三美湾度假酒店、国家开发银行发展研究院等项目；在鹿回头半岛，要继续推进新佳旅业鹿回头项目、沃尔沃帆船赛经停港基地等项目建设。

（四）着力打造特色旅游产品体系。积极引进国内外有实力的大型旅游企业，大力开发海上游、海上垂钓、海上运动、海底观光、潜水等海洋旅游系列产品，规划建设海洋、演艺等主题公园。积极促进三亚邮轮母港建设和邮轮旅游发展，推动编制三亚市游艇产业发展战略规划以及产业布局规划，培育游艇产业。充分利用气候优势和温泉、森林等资源，积极开发体育休闲项目，规范发展高尔夫旅游，加快发展康体保健、森林生态旅游，抓好槟榔村5A级乡村旅游区建设，积极发展红色旅游，形成以海洋旅游为中心、种类更加丰富的特色产品体系。

（五）全面提升旅游管理和服务水平。增强品牌意识，加强旅游品牌培育和保护。有序推进旅行社、导游管理体制改革，积极引进知名国际旅行社在三亚注册。加快打造旅游公共服务平台，全力推进游客到访中心和“天涯通”旅游信息服务系统建设，全面改造旅游标识系统等服务设施。认真开展“旅游品质服务年”和旅游服务标准化工作，进一步加强从业人员培训，建立健全旅游行业诚信体系、旅游安全预警和应急机制。大力开展旅游环境综合整治，严厉打击黑车黑导、摩托车非法营运、欺客宰客、“两抢一盗”等行为。以三亚湾、大东海等区域为重点，建设完善休闲广场、人行道、停车场、隔离带、垃圾箱、公共厕所等设施，坚持集中整治与长效管理相结合，突出治理环境卫生脏乱差、交通秩序混乱、占道经营摆摊等问题。

（六）进一步加强国内外市场开发。积极参与筹办首届博鳌国际旅游论坛，继续在主流媒体投放三亚旅游宣传广告，树立城市整体形象。加大旅游营销投入，建立旅游营销系统，积极整合旅游协会、航空、旅游企业和媒体的力量，充分利用新调整的免签证等出入境管理政策，有针对性地做好俄罗斯市场恢复、印度市场开拓、香港“一程多站”深化合作、西欧及北欧市场深入开发、中亚及西亚市场突破等工作。充分利用上海世博会海南周活动等平台，加强国内外市场宣传促销。认真分析评估宣传促销活动成效，着力提升宣传促销工作质量。

二、保持投资稳定高效增长，进一步扩大居民消费需求

要准确把握中央和省里的投资政策与导向，进一步加大投资力度，优化投资结构。同时，要将增加居民消费作为扩大内需的重点，着力增强消费对经济发展的拉动作用。

（一）保持投资稳定高效增长。紧紧围绕国家、省的产业政策和投资导向，找准项目与政策的对接点，积极争取国家、省投资项目，抓好新增中央投资项目建设。依托生态环境优势和国际旅游岛建设，以海棠湾国家海岸、崖城创意产业基地和海上旅游为重点，策划和储备一批项目，做好前期工作。大力推进投融资体制改革，认真实施《三亚市一会五库管理规定》、《三亚市政府投资建设项目代建制管理办法》等规定，强化政府投资项目管理。积极开展2009年促进经济平稳较快发展各项政策的效果评估并进行调整完善，进一步优化投资发展环境，引导和带动社会投资。今年我市安排重点项目83个，要加大征地拆迁工作力度，坚决打击违法建设、非法买卖集体土地等行为，严格实行重点项目跟踪服务，强化项目业主责任，确保项目按时推进。要加强工程建设监理和施工安全管理，确保项目安全生产和建设质量。

（二）进一步扩大居民消费需求。积极促进就业，适当增加企事业单位职工的工资性收入，进一步提高最低工资标准，通过实施各项补贴、征地补偿等政策，增加城乡居民经营性收入、转移性收入和财产性收入，着力提高低收入群体的收入水平和中等收入者的比重，增强居民消费能力。继续加快商贸基础设施建设，积极推进第一市场和金鸡岭等农贸市场升级改造，支持国内外品牌大型连锁零售企业进入我市。加快推进家政服务体系建设，积极发展服务性消费。大力实施“万村千乡市场工程”和新农村现代流通网络工程，进一步做好家电、农机、汽车下乡以及汽车以旧换新等政策，扩大农村消费规模。

三、加快产业结构调整，打造特色产业体系

要在提升旅游产业发展质量的同时，继续打造具有三亚特色的产业体系，促进经济结构调整取得新进展。

（一）引导房地产业平稳可持续发展。进一步加强土地资源管理和调控，坚持实行国有土地使用权“招拍挂”出让制度，严格控制规划指标。按照“高端、适度、有序”的原则，鼓励开发高品质的星级度假宾馆、度假村等项目，稳步发展适应御寒、疗养等不同需求、富有三亚特色的度假居住型房地产。加强产权式度假酒店开发建设和管理，推动房地产中介市场健康发展，进一步规范家庭旅馆经营和房屋租赁管理。着力加强房地产市场监控，规范商品房预售管理，建立健全企业信用制度，依法严厉查处囤积土地、捂盘惜售、哄抬价格等行为，抑制房地产投机炒作。

（二）积极推进商贸产业等服务业发展。在扩大城乡居民消费需求的同时，立足旅游城市的特点，结合国际旅游岛建设，大力开发地方特色旅游商品，积极支持专业公司建设、经营免税店，加快完善休闲度假区的商业配套设施，努力增强旅游商业网络服务功能。大力规范消费市场秩序，营造良好的消费环境。同时，加快推进交通运输、仓储物流、金融保险、邮电通讯、信息咨询等现代服务业发展，不断完善旅游城市综合服务功能。

（三）加快优势特色农业发展。进一步完善农产品生产布局规划，积极支持冬季瓜菜、热带水果、热带花卉、热带经济作物以及畜产品等特色现代农业园区发展，支持南繁育种基地建设。以“设施农业+产业化”为重要抓手，推动农业现代化进程。继续鼓励和支持社会力量建设设施农业标准化基地，支持农民专业合作社发展，支持龙头企业、农民专业合作社和种养大户实行标准化生产，大力推动兰花、玫瑰等基地建设。进一步完善农村科技服务体系，抓好农技推广、培训、咨询等工作。加快推进农产品加工业发展，拓展农业产业链。不断强化农产品质量安全保障工作，树立无公害农产品品牌。进一步完善三亚农业信息网以及12316、12582服务热线，积极推进海棠湾农产品综合批发市场、崖城蔬果储存销售运输中心建设，完善农产品市场销售体系和运行机制，做好农产品市场信息和流通服务。

（四）大力发展海洋经济。完成《三亚市海洋功能区划》修编，进一步完善和推行海域使用权公开招标拍卖制度，有序推进东锣岛、西鼓岛等无居民海岛开发。积极推进崖州中心渔港建设，继续支持渔船更新改造，实施渔业综合补给船项目，建立健全渔业长效服务保障机制，采取“企业+渔民”的方式，开发西中沙等外海渔业资源。加强以人工鱼礁与增殖放流为主的海洋牧场规划建设，满足休闲渔业和游钓业市场需求。积极引进优质水产品种，推广标准化养殖，加快推进无公害水产品产地和产品认证工作，扶持水产品加工业发展。支持中海油、中石油建设南海油气资源勘探开发服务基地。

（五）继续扶持创意产业发展。加快创意产业园征地拆迁和基础设施建设，实施项目跟踪服务，全力确保落户项目按时推进。进一步完善和落实项目跟进投资、风险投资、贷款贴息、财政扶持补贴以及生活服务区用地和生产生活用房等优惠政策，尽快形成适合创意产业发展的人才培训、生产创作、市场运营环境。积极推进综合孵化器和标准厂房设计与建设，为企业入园提供支持。进一步加强宣传和招商工作，充分发挥优势项目的吸引和带动作用，引进有实力的创意产业特别是太阳能、电子信息类产业项目。

（六）着力培育文化体育产业。加快完善文体基础设施，完成三亚市体育中心建设并投入使用，积极策划推进市群众艺术馆、三亚大剧院、市民文化广场等项目。进一步加大文化体制改革力度，继续引进有实力的文化产业项目。积极策划一批具有三亚文化特色的项目，重点扶持推出一台大型文艺演出节目。进一步加强黎族等少数民族文化的挖掘、开发、保护和传承，积极推进崖城历史文化名镇古城修缮等八个改造项目，策划打造中国“衣食父母”主题公园。在办好美丽经济系列赛事、环岛国际公路自行车赛等文体赛事的同时，创造条件建设国家体育冬训基地，引进和培育品牌会展，支持有实力的团体赴国内外参加一些有影响的比赛和演出。积极建立健全公共文化服务平台，加强市图书馆建设管理，加快完善两区六镇文体配套设施，继续实施农家书屋、广播电视村村通等文化惠民工程，积极开展社区文化活动、乡村文化活动、文化下乡活动、全民健身活动，丰富城乡居民的文体生活，营造浓厚的文化氛围。

四、进一步深化经济领域改革，不断提高对外开放水平

在国际旅游岛建设的新形势下，要继续推进经济领域各项改革，着力提高对外开放水平，增强发展活力和动力。

（一）努力优化经济发展环境。积极推进市属国有企业调整重组，完成59家国企关闭退出工作。不断完善国企产权管理、重大事项管理、财务监督管理、业绩考核管理和企业风险管理，建立健全现代企业制度，培育国企上市主体，提升国企效益。继续实施中小企业成长工程，支持中小企业解决融资难问题。鼓励、引导行业协会和中介组织规范发展，努力完善市场功能和服务。进一步优化财政支出结构，加强政府预算编制和预算执行管理，加快发展金融保险业，加大财政金融支持经济发展的力度。

（二）进一步提高对外开放水平。精心组织参加“珠洽会”、“厦交会”、香港商贸推介会以及上海世博会海南周等活动，力争在整体形象宣传、经济交流合作、项目招商以及旅游、房地产专场推介上取得新成果。努力提升招商质量，以城市和产业发展规划为指导，着力引进投资规模大、技术含量高、产业带动力强的企业与项目。继续实施科技兴贸战略，重点发展海产品、热带花卉、瓜果等热带优势农副产品及其深加工产品，鼓励具有自主知识产权、自主品牌的产品出口。加强口岸基础设施建设，充分利用凤凰国际机场、南山货运港平台，进一步提升口岸服务水平，优化外贸发展环境。

五、加强生态文明建设，增强可持续发展能力

要保持和利用好三亚的生态环境优势，进一步强化节能减排工作，加强生态环境保护，努力实现可持续发展。

（一）切实抓好节能减排工作。加快推进田独、红塘湾、海棠湾、荔枝沟落笔洞区等污水处理工程、城市污水管网改造工程、城市生活垃圾收集转运工程、区镇垃圾中转站、社区和村级垃圾收集点以及亚龙湾区域集中供冷节能工程等项目建设。进一步提高建设项目环评效率，切实落实节能减排政策，认真实施差别电价等措施，进一步完善污水处理费收费标准。大力发展新型清洁能源和材料，引进和支持使用太阳能产品，启动绿色环保节能型酒店科技示范工程，加快实施节能产品惠民工程，鼓励使用建筑节能技术。完成农村“一池三改”户用沼气池建设2100户，继续实施“新农村新科技集成示范”项目，加快推进再生资源回收体系建设和管理，推动循环经济发展。

（二）加强生态保护和环境治理。启动三亚生态市建设，积极开展创建国家环境保护模范城市工作。大力推进饮用水水源保护区规划建设，继续抓好造林绿化、护林防火、红树林湿地保护等工作，严厉查处各类非法毁林案件。加强海洋环境监察，继续开展“蓝丝带”海洋环境保护，强化重点海湾海洋环境和海岸工程监管，抓好综合治理三亚湾人工恢复岸滩工程项目建设。加快实施三亚西河（槟榔河续建）综合整治工程，做好防洪保安工作。积极开展环保专项行动，加大对噪声、尾气等污染的治理力度，继续保持良好的生态环境。

六、积极统筹城乡发展，改善居民生活条件

目前，三亚的城市功能和管理水平与国际旅游岛建设的要求还有较大差距。要大力加强城市建设，完善城市功能，优化城市环境，同时进一步统筹城乡发展，继续推进新农村建设。

（一）大力加强基础设施建设。在东部，继续推进海棠湾基础设施建设，强化海棠湾片区集聚能力。在西部，稳步推进崖城创意产业基地基础设施建设，提高园区配套水平。在北部，加快绕城高速公路及相关连接线建设，尽快打通东西高速路通道。在中心城区及周边，加快完成城市综合交通规划、老城区综合交通整治规划，继续推进凤凰镇至西线高速公路道路、月川新城区道路等工程建设，尽早开工新风桥、三亚桥重建工程、榆亚大道改建工程、长途公交汽车站搬迁工程、火车站出口道路及场站工程，加快完善停车场等设施，努力构建畅通便捷的交通网络体系。此外，要尽快编制完成《三亚市城乡公共交通一体化规划》，积极推进农村客运网络化、公交化和城乡道路运输一体化建设。

（二）加快旧城改造步伐。积极推进榕根、月川、阳光海岸、海坡村等旧城区改造，整合利用旧城土地资源，提升城市建设水平。加强规划管理，健全和优化路网结构，结合旧城改造工作全面完善城区交通、水电、燃气、通讯、商业、教育、医疗等设施配套，打造“半小时便利生活圈”。推动旧城改造主体下移，由市政府制定规划、建章立制、统筹指导，由各区、镇具体负责实施，充分发挥村（居）委会和村民股份制公司的作用。坚持以人为本，在一定规划区域内实行回迁安置，并为被拆迁群众提供经营性用房，建立健全被拆迁群众就业、社保、教育和特困户、低保户扶持等配套政策体系，解决其长期生活和发展问题。

（三）进一步提升城市管理水平。加快推进信息化基础设施建设，探索加强规划、建设、综合执法、旅游、公安等部门之间的信息互通与共享，为充分发挥信息技术在城市管理中的作用创造条件。进一步完善城市“网格化”管理，建立健全城管日常巡查制度和精细化监督考评机制。严格落实属地管理责任，加强社区建设，强化居委会的作用，不断完善城市基层管理和服务体制。积极探索推行疏堵结合的管理方式，对餐饮、水果等商贩实行规范化改造，完善停车场、垃圾箱、公共厕所等配套设施。强化部门联合执法，大力开展违法抢建抢种现象排查治理，切实加强交通道路运输行业管理，持续开展交通秩序、市容管理等专项执法整治。加强行政执法人员培训和管理，切实做到文明执法、依法行政。认真落实三亚市国家卫生城市长效管理机制和细则，不断完善和认真实施六大长效管理机制，巩固创卫和城市管理成果。

（四）继续推进新农村建设。着力加快大隆灌区工程、病险水库加固工程等水利设施工程建设，继续实施农村公路通畅工程，抓好饮水安全、改水改厕、垃圾集中处理以及老区建设、扶贫攻坚等工作，完成250个村民小组的文明生态村创建，不断改善农村生产生活环境。鼓励乡镇企业向园区、城镇集中，促进农村二、三产业发展。以实施“阳光工程”为重点，积极开展农村劳动力技能培训，增强农民转产转岗就业能力，提升农民整体素质，实现农村富余劳动力转移就业1万人的目标。进一步深化农村综合配套改革，积极开展农村土地承包管理信息网络系统建设，妥善处理土地纠纷等问题，尽快完成林改任务，继续完善小额信贷、农户联保贷款和农业保险等金融服务。全面推行惠民“一卡通”改革，全面完成农村义务教育管理体制改革等工作。进一步巩固和深化农垦改革成果，促进农垦发展。

七、大力发展社会事业，打造三亚人民的幸福家园

改善民生是发展的根本目的。要加快实施《三亚市2008―2013年重点民生项目发展规划》，推动社会事业发展，让城乡居民得到更多实惠。

（一）着力改善群众住房条件。进一步加大保障性住房建设力度，从土地供应、规划条件、建设资金、政策扶持等方面予以支持，确保城乡居民住房条件有明显改善。着力解决低收入群众、国企职工、包括乡镇七所八站在内的事业单位以及党政机关干部职工的住房困难问题。今年要新建经济适用房1500套、“双限房”2800套、公共租赁房300套，要确保建设质量，确保保障性住房满足需求。严把保障性住房资格审核关，确保分配公平合理。进一步提高补助标准，加快推进农村危房和简易住房改造工作。

（二）认真开展就业社保工作。进一步加强就业服务信息网络建设，大力开展就业培训和就业扶助，着力抓好高校毕业生、下岗职工、农民工和困难群体就业工作。积极创建“国家级创业型城市”，以创业带动就业。加快推进城镇老年居民养老保险和新型农村社会养老保险工作，发展多层次的社会化养老服务。认真落实《三亚市城乡居民基本医疗保险暂行办法》，实现应保尽保。进一步提高城乡低保补贴标准，继续推进城乡医疗救助工作，提高社会救助、社会福利综合服务能力。不断完善价格监测体系，加强涉及民生的价格监管。

（三）积极发展教育事业。加快中小学布局调整，实施学校标准化建设，确保完成90%的中小学校舍改危工作任务。通过实施“引培工程”等方式加强教师队伍建设，提升教育教学质量。加快发展高中阶段教育，继续扩大优质高中教育规模。不断优化中职学校专业设置，开展“订单式”人才培养，拓宽学生就业渠道。进一步规范学生资助体系管理，抓好“两免一补”、“三免一补”等工作。支持高等教育发展，继续推进琼州学院三亚校区、理工职业学院、城市职业学院等项目，加强特色学科和专业建设，进一步健全人才引进和培养机制，支持各高校与国内外知名院校加强交流合作。

（四）提高医疗卫生服务水平。积极推进市人民医院改扩建二期工程、中医院二、三期建设工程，加快解放军总医院海南分院项目建设。进一步加强社区卫生服务中心、服务站和农村卫生院建设，健全城乡基层医疗卫生服务体系。启动并认真实施国家重大和基本公共卫生服务项目。认真落实近期重点实施方案，加快推进医药卫生体制改革。继续加强食品药品监管，实施食品药品放心工程。进一步完善计生公共服务体系，健全人口计生利益导向机制，稳定低生育水平。

（五）切实维护社会和谐稳定。继续推进“法律六进”工程，积极开展社会主义精神文明建设。进一步完善社会治安防控体系，加强社会治安综合治理，深入开展打击“两抢一盗”和毒品犯罪等专项行动，严密防控和严厉打击各种犯罪活动。切实落实安全生产责任制，进一步强化重点领域专项整治，坚决遏制重特大安全事故的发生。抓好矛盾纠纷排查和重信重访专项治理，加强信访事项督查督办，充分发挥基层政府和组织的管理作用，有效预防和妥善处置群体性事件。建立健全重大公共安全事件预警、应急、救援体系，增强政府应对突发事件的能力。

（六）大力发展其它社会事业。积极支持中央和省驻三亚单位充分履行职责。广泛开展国防教育，支持国防建设，进一步加强双拥共建、国防动员和民兵预备役工作。加快发展妇女儿童、老龄、红十字、慈善和残疾人事业，继续做好新闻出版、民族宗教、统计、外事、侨务、气象、防震减灾、档案、地方志等工作。

八、以廉洁高效为目标，加强政府自身建设

今年的工作任务十分艰巨。要继续推动体制机制创新，加强政府自身建设，不断提高行政效能。

（一）进一步加强民主法制建设。严格执行市人大及其常委会的决议、决定，定期向市人大及其常委会报告工作，向市政协通报政务情况，认真做好人大议案、建议和政协提案的办理工作，自觉接受人大法律监督、工作监督和政协民主监督。积极加强同各民主党派、工商联以及无党派人士的联系，认真听取他们的意见。支持工会、共青团、妇联等人民团体依法参与社会管理和公共服务，充分发挥其桥梁纽带作用。进一步完善政务公开工作，增加行政权力运行的透明度，切实保障人民群众的知情权、参与权、表达权和监督权。

（二）努力提高行政效能。按照精简、统一、高效的原则，继续深化市政府机构改革。全面启动政务服务中心，积极推行并不断完善“集中办公、统一受理、并联审批、联席会议、限时办结”的运行机制。进一步理顺海棠湾审批权限，提高审批效率。加快推进12345政府服务热线和全市政务信息处理平台建设，加强信息资源共享。进一步加强公务员队伍建设，积极推进层级管理，层层落实工作责任，强化督查督办。进一步完善机关效能告诫办法、行政过错责任追究办法，严格落实各项问责制度，把群众举报和媒体曝光作为查办行政低效的重要线索，一经查实，严厉追究，绝不姑息。全面完成镇机构改革、镇财政管理体制改革等工作，进一步加强基层政府、机构和组织建设，不断完善工作奖励激励机制，充分调动基层干部的工作积极性。以加快社会服务体系建设为重点，全面推进事业单位改革。

（三）不断强化政府廉政建设。认真开展廉政教育，着力降低行政运行成本，坚决制止奢侈浪费行为。积极建立健全各项制度，进一步深化源头治腐工作，严格执行行政机关“一把手”不得直接分管本单位工程项目和财务的规定。加大行政监察和审计监督力度，着力规范行政审批、执法检查、收费征税、行政强制、立案处罚等行政权力的运作规程，认真治理城市规划、土地管理、征地拆迁、工程建设、交通运输、税收征管等领域的突出问题。积极开展反腐倡廉“制度执行巩固年”活动，继续保持惩治腐败高压态势，严肃查处各类违法违纪案件，坚决纠正各种不正之风。

各位代表，2010年是实施“十一五”规划的最后一年，也是国际旅游岛建设的开局之年，任务繁重，使命光荣。让我们在省委、省政府和市委的坚强领导下，深入贯彻落实科学发展观，团结一致、开拓创新，为促进三亚又好又快发展、推动国际旅游岛建设而努力奋斗！
